# Reviewer Acknowledgments

**DOI:** 10.1002/mlf2.12162

**Published:** 2025-01-28

mLife thanks the following reviewers for their great support and contribution in 2024.

Max Amenabar

Kristine Arnvig

Rohan Balakrishnan

Stephen L. Bearne

Ivan Berg

Shuangyu Bi

Daniela Billi

William Brazelton

Dongbo Bu

Shang Cai

Zhao Cai

Pengbo Cao

Eric Caragata

Hongliang Chai

Subhadeep Chatterjee

Xuefeng Chen

Yan Chen

Yaoqing Chen

Yunru Chen

Zheng Chen

Parkson Lee‐Gau Chong

Alessandro Concas

Lei Dai

Ajai A. Dandekar

Xin Deng

Ye Deng

Yinyue Deng

Chen Ding

Feng Ding

Tao Dong

Xiyang Dong

Hong Du

Lei Du

Shishen Du

Wenbin Du

Chengguo Duan

Xiangke Duan

Abdelaziz Ed‐Dra

Yupeng Fan

Jie Feng

Ailing Fu

Danny Fung

Yunn‐Hwen Gan

Haichun Gao

Renjun Gao

Xiang Gao

Zheng Gao

Fernando García Navarro

Luyao Gong

Yanhai Gong

Xin Guo

Xue Guo

Yunxue Guo

Lin Han

Wenyuan Han

Yiping Han

Ding He

Hisashi Hemmi

Wolfgang Hess

Yuzhi Hong

Panpan Hou

Anyi Hu

Jiao Hu

Song nian Hu

Xiaoke Hu

Xiaomin Hu

Shi Huang

Xing Huang

Ying Huang

Anthony Huffman

Dafeng Hui

Quanjiang Ji

Ning Jia

Na Jia

Xinmiao Jia

Yantao Jia

Zhongjun Jia

Huahua Jian

Huifeng Jiang

Tom Kieft

Mart Krupovic

Tomoko Kubori

Andrey Kulbachinskiy

Maggie Lau Vetter

Shuai Le

Caoqi Lei

Fumin Lei

Bing Li

Fu‐Li Li

Ruoyu Li

Yong Li

Yuqing Li

Zhiyuan Li

Haihua Liang

Hao Lin

Steven Lindow

Bennjamin Liu

Chang Liu

Hongfang Liu

Jiwen Liu

Junyan Liu

Qian Liu

Suo Liu

Xiao Liu

Xing Liu

Yajun Liu

Wen‐Yong Lou

Fengmin Lu

Ling Lu

Bo Ma

Luyan Ma

Yingfei Ma

Diana Machado

Yuxin Mao

Paraskevi Mara

Prabina K. Meher

Alessio Mengoni

Zohreh Moradi

Da‐Shuai Mu

Yong Nie

Kang Ning

Guohui Pan

Sheo Shankar Pandey

Petra Patakova

Tong‐Tong Pei

Xu Peng

Maria‐Cristina da S. Pranchevicius

David Prangishvili

Sarah P. Preheim

Meng‐Chun Qi

Guoliang Qian

Wei Qian

Wei Qin

Jiguo Qiu

Zhe‐Xue Quan

Jasna Rakonjac

Tiago Ramalho

Giordano Rampioni

Eric Rubin

Hossein Samadi Kafil

Xiaolong Shao

Zongze Shao

Qunxin She

Qingtao Shen

Xihui Shen

Yulong Shen

Guiyang Shi

Wenyu Shi

Patrick O. Sorensen

Kenneth Stedman

Chang Su

Zhoutong Sun

Jiufeng Sun

Yanni Sun

Jean Swings

Liang Tao

Meifeng Tao

Pan Tao

Xuanyu Tao

Yong Tian

Santangelo Tom

Bo Wang

Fang Wang

Jianjun Wang

Jinfeng Wang

Jufang Wang

Jun Wang

Kai Wang

Pei Wang

Ruojun Wang

Wen Wang

Xiaoxue Wang

Yingcheng Wang

Zhong Wei

Steve Whyard

Chenggang Wu

Chongming Wu

Yang Wu

Chuanwu Xi

Xingjia Xiang

Genhui Xiao

Liwen Xiao

Jianping Xie

Jinling Xu

Ying Xu

Huilin Yan

Qing Yan

Zhen Yan

Jianyi Yang

Jun Yang

Liang Yang

Sidi Yang

Yiling Yang

Yunfeng Yang

Fangyou Yu

Jin Yu

Yunsong Yu

Junhua Yuan

Jin Zeng

Cheng‐Cai Zhang

Ge Zhang

Guijun Zhang

Guoliang Zhang

Jing‐Ren Zhang

Qiong Zhang

Rui Zhang

Xiaohua Zhang

Yanfei Zhang

Yongyu Zhang

Yu Zhang

Yu‐Zhong Zhang

Zheng Zhang

Rui Zhao

Yi‐Lei Zhao

Beiwen Zheng

Yue Zheng

Weihong Zhong

Jingwen Zhou

Peng Zhou

Yaoqi Zhou

Yongjin Zhou

Zhemin Zhou

Zhihua Zhou

Yan Zhu

